# Antibiotic prescription in bone augmentation and dental implant procedures: a multi-center study

**DOI:** 10.1186/s12903-023-03534-6

**Published:** 2023-10-29

**Authors:** Nikoo Bazsefidpay, Fredrik Holmqvist, Dalia Khalil, Cecilia Larsson Wexell, Margareta Hultin, Peter Nilsson, Bodil Lund

**Affiliations:** 1 Head-neck and plastic surgery clinic, Department of Oral and Maxillofacial Surgery, Örebro University Hospital, Örebro, Sweden; 2 School of Medical Sciences, Örebro University, Örebro, Sweden; 3 Department of Dental Medicine, Division of Orofacial Diagnostics and Surgery, Karolinska Institutet, Huddinge, Sweden; 4 Department of Dental Medicine, Division of Oral and Maxillofacial Surgery, Jönköping, Sweden; 5 Department of Dentistry, King Fahad General Hospital, Jeddah, Saudi Arabia; 6 Department of Oral and Maxillofacial Surgery, Skåne University Hospital, Lund, Sweden; 7 Department of Biomaterials, Sahlgrenska Academy, University of Gothenburg, Gothenburg, Sweden; 8 Department of Oral and Maxillofacial Surgery and Oral Medicine, Malmö University, Malmö, Sweden; 9 Department of Dental Medicine, Division of Periodontology, Karolinska Institutet, Solna, Alfred Nobels allé 8, 141 04 Sweden; 10 Medical Unit of Plastic Surgery and Oral and Maxillofacial Surgery, Department for Oral and Maxillofacial Surgery and Jaw Orthopedics, Karolinska University Hospital, Stockholm, Sweden

**Keywords:** Antibiotic prophylaxis, Bone augmentation, Dental implant insertion, Infection, Guidelines

## Abstract

**Background:**

Adherence to antibiotic recommendations and safety aspects of restrictive use are important components when combating antibiotic resistance. The primary aim of this study was to assess the impact of national guidelines on antibiotic prescriptions for bone augmentation procedures among dentists working at three specialized clinics. The secondary aim was to assess the occurrence of postoperative infections.

**Methods:**

Medical charts of 400 patients treated with bone augmentation were reviewed: 200 in the years 2010–2011 and 200 in 2014–2015. The Swedish national recommendations for antibiotic prophylaxis were published in 2012.

**Results:**

There was a wide variation in antibiotic regiments prescribed throughout the study. The number of patients treated with antibiotic prophylaxis in a single dose of 2 g amoxicillin, and treated as advocated in the national recommendations, was low and decreasing between the two time periods from 25% (n = 50/200) in 2010–2011 to 18.5% (n = 37/200) in 2014–2015. The number of patients not given any antibiotics either as a prophylactic single dose or during the postoperative phase increased (*P* < 0.001). The administration of a 3-7-days antibiotic prescription increased significantly from 25.5% in 2010–2011 to 35% in 2014–2015. The postoperative infection rates (4.5% and 6.5%) were without difference between the studied periods. Smoking and omitted antibiotic prophylaxis significantly increased the risk of postoperative infection. Logistic regression analyses showed that patient male gender and suffering from a disease were predictive factors for the clinician to adhere to the guidelines.

**Conclusions:**

After introduction of national recommendations for antibiotic prophylaxis before bone augmentation procedures, the patient group receiving a single preoperative dose decreased while the group not given antibiotic prophylaxis increased. There was no difference in occurrence of postoperative infections between the two time periods. The results indicate a need for educational efforts and strategies for implementation of antibiotic prudence and awareness among surgeons performing bone augmentation procedures.

## Background

Antimicrobial resistance is an alarming and increasing problem world-wide seriously threatening modern health care where many treatment options are dependent on the availability of effective antibiotics [1, 2]. The emergence of reduced susceptibility to antibiotics is strongly correlated to the total consumption [3]. Important measures for combating this problem are prevention of misuse and overuse by implementing guidelines and counteracting nosocomial transmission of resistant microorganisms. To minimize the antibiotic use, the indications for antibiotic prophylaxis and treatments needs to be assessed in different clinical situations. One such area of antibiotic utilization is prophylaxis during bone augmentation procedures prior to or in conjunction with dental implant surgery. Currently there is a lack of controlled clinical studies concerning antibiotic prescription patterns among health care professionals, including the clinical efficacy of systemic antibiotics on reducing surgical site infection, and putative adverse effects.

Patients’ expectations on the treatment with fixed prosthetic replacements are very high. Anatomic defects and lack of bone volume makes bone-grafting procedures frequently necessary. In jaws resorbed after trauma, or infection, it may be necessary to augment the alveolar bone to enable dental implant rehabilitation. In general, there are a variety of techniques to augment jawbone with arbitrary recommendations for the duration of post-extraction healing. Autografts are harvested and transferred within an individual from e.g. mandibular ramus, chin, or iliac crest, and commonly collected with a bone-scraper, mini-saw, or drill. Commercially available bone substitutes may be from cadaver bone, human and animal origin, or synthetically produced. Generally cadaver bone is the most used bone substitute today and mineralized freeze-dried bone allografts and xenografts functions well where the anatomical shape must be restored since the substitutes do not resorb. Many different synthetic bone substitute materials are tailor-made and available with different chemical and physical properties to meet market demands regarding resorption rate, chemical composition, granule size and degree of porosity. In addition, thin resorbable or non-resorbable synthetic or xenograft membranes are sometimes used to cover the augmented bone site under the mucosa to keep the bone in place and prevent ingrowth of soft tissue into the area.

Although it is well known that the addition of a foreign or grafted material into a surgical site increases the risk of postoperative infection, reports regarding postoperative infections after these interventions are essentially lacking [4–7]. Empiric antibiotic prophylaxis has a central role in most of the surgical bone augmentation procedures. Upon introduction of these interventions, antibiotic treatment for approximately 10 days was generally considered appropriate. There is however no scientific evidence for this antimicrobial precaution [8, 9]. In Sweden, there were no guidelines for antibiotic prescription in conjunction with bone augmentation procedure until 2012. Swedish national guidelines for antibiotic prophylaxis in dentistry were published to dissuade from prolonged prophylactic antibiotic utilization. Instead a single dose of 2 g amoxicillin 1 h preoperatively, or 600 mg clindamycin in case of allergy, was suggested as sufficient protection [10]. Although numerous studies show no further benefit of an extended antibiotic prophylaxis beyond the day of surgery for other surgical procedures, the scientific evidence is sparse regarding implant dentistry [8, 9, 11, 12]. Since bone augmentation is a common therapy in dentoalveolar surgery, the amount of antibiotics used on this indication in otherwise healthy patients may pose a significant contribution to the antibiotic consumption in dentistry.

Since the development of antibiotic resistance is considered the greatest threat to modern health care any effort to reduce unnecessary utilization, such as identifying areas of improvement, is of outmost importance. The aims of the current study were primarily to investigate antibiotic prescription pattern among Swedish dentists during bone augmentation procedures and to study the effect of published guidelines on the antibiotic prescription pattern. A secondary aim was to investigate the effect of antibiotic prophylaxis use on occurrence of post-operative infection after bone augmentation procedures.

## Methods

### Study design

A retrospective cross-sectional patient record study, to review the utilization of antibiotic prophylaxis, was performed at four different Swedish clinics performing bone augmentation procedures in conjunction to dental implant treatment. Two time periods were studied, before and after the publication of the Swedish national recommendations on antibiotic prophylaxis in 2012, in order to scrutinize the influence of the recommendations on the prescription behavior. The four specialized clinics performing implant surgery included were the Department of Periodontology at the Odontology Institution in Jönköping, the Department of Oral and Maxillofacial Surgery at the Odontology Institution in Jönköping, the Department of Oral and Maxillofacial Surgery at the Eastman Institute, Stockholm, and the Department of Oral and Maxillofacial Surgery at South Älvsborgs Hospital, Borås. A power calculation based on the assumption that 80% of the patients received prolonged antibiotic prophylaxis before the publication of the recommendations and 60% afterwards, gave a total required sample size of 200 patients at power 80% and alfa 0.05. Since the sample size calculation was based on a crude estimation, it was decided to double the number of included cases. Prior to onset, the study was approved by the Regional Ethics Committee, Stockholm (Ref no 2016/609 − 31). The study was performed in compliance with the STROBE statement checklist (https://www.strobe-statment.org).

### Data collection

A total of 400 medical charts were reviewed: 200 from the periods January 1st 2010, to December 31st, 2011 and 200 from January 1st, 2014 to December 31st, 2015. Each clinic contributed with 100 cases, 50 from each time period. The criterion for inclusion was intraoral bone augmentation procedures performed on the indication of insufficient bone volume for immediate or later placement of dental implants to replace one or several missing teeth. Exclusion criteria were age below 18 and incomplete or missing patient medical charts. If more than one augmentation procedure was done on the same patient and site, the first intervention was selected and registered. Patients were identified using a computerized search tool on treatment codes. Data was collected regarding patient characteristics such as general health, medications, allergies, smoking habits, age, gender, exposure to radiation therapy as well as the surgeon’s type of training and education. Also, data were registered regarding the local diagnose motivating treatment, type of surgical procedure, prescription of antibiotics, type of compound, dose and duration, choice of material used for bone augmentation and if an implant was inserted at the same time. In patients with antibiotic allergies, type of replacement compound, given dose and duration was noted. All postoperative infections occurring during the first three months after the surgery were registered as well as how they were managed. Data collection was done using a standardized case record form and the extracted data was anonymously coded to ensure patient integrity.

### Demographic data of patient population

From a total of 200 patient records included in 2010–2011, there were 55.0% (n = 110) males and 45.0% (n = 90) females, and in 2014–2015 there were 51.5% (n = 103) males and 48.5% (n = 97) females. In 2010–2011, 51% (n = 102) and 51.5% (n = 103) in 2014–2015 of the total patients were healthy with no medication. The rest of the patients were diagnosed with various diseases, most commonly high blood pressure, asthma, depression, hypothyroidism, or migraine. In 2010–2011 there was no entry in 30% (n = 60) of the patient records regarding smoking habits, the corresponding figure for 2014–2015 was 37.5% (n = 75). 23.5% (n = 47) of the patients were stated to be smokers in 2010–2011 and 16% (n = 32) in 2014–2015. Two patients were on bisphosphonate treatment in 2010–2011 and one in 2014–2015. No patient exposed to radiation therapy was treated with bone augmentation in neither time periods. There were no significant differences between the two time periods regarding gender, health status and smoking habits. The number of patients in the category 17–30 years old decreased between the two time periods while the distribution of other age groups was unchanged (*P* = 0.012). The demographic data of patients is summarized in Table 1.

**Table 1:** Demographic data of included patients during the two study periods

Characteristics	2010–2011 n = 200 N (%)	2014–2015 n = 200 N (%)	Chi-square	*P*-value
Sex				
Male Female	110 (55) 90 (45)	103 (51.5) 97 (48.5)	0.49	0.48
Age (years)				
17–30	51 (25.5)	30 (15)	6.19	0.012
31–40	9 (4.5)	8 (4)	0.06	0.80
41–50	18 (9)	25 (12.5)	1.28	0.25
51–60	34 (17)	37 (18.5)	0.15	0.69
61–70	65 (32.5)	64 (32)	0.01	0.91
> 70	23 (11.5)	36 (18)		
Smoking habit				
Smoker	47 (23.5)	32 (16)	3.55	0.059
Non-smoker	93 (46.5)	93 (46.5)	0.002	0.99
Not stated	60 (30)	75 (37.5)	2.52	0.117
Implant installation protocol				
Delayed implant installation^a^	87 (43.5)	127 (63.5)	16.08	0.001
Simultaneously with bone augmentation^b^				
One stage technique^c^	23 (11.5)	12 (6)	3.79	0.051
Two stage technique^c^	90 (45)	61 (30.5)	8.95	0.002

Abbreviation: n, number of patients

^a^Implant installation 3–6 months after bone augmentation

^b^Implant installation and bone augmentation in same session

^c^One stage technique refers to implant installation and abutment connection in one stage, while two stage technique refers to implant installation first followed by abutment connection in a second stage

### Summary of clinical procedures

The implants in combination with bone augmentation procedures were installed either in the same surgical session (one or two staged implant surgical protocols), or after a healing period, i.e. not on the day of the bone graft procedure (Table 1-Summary of clinical procedures). In 2010–2011 49.5% (n = 99) of the patients underwent an autograft bone augmentation, 33.5% (n = 67) xenograft, 11.5% (n = 23) a mixture of them and 5.5% (n = 11) other procedures for example sinus lift, and in 2014–2015 the corresponding number was 43.5% (n = 87) autograft, 42.5% (n = 85) xenograft, 12% (n = 24) a mixture and 2% (n = 4) other procedures. Most of the autograft procedures had bone scraped locally or bone harvested from zygomatic bone, mandibular ramus, nasal spine, or iliac crest. During bone augmentation surgery, sinus lift procedures were performed in 19% or 16% of the cases in 2010–2011 and 2014–2015, respectively. In 2010–2011, 26.5% (n = 53) of the cases involved placing resorbable membranes during surgery, while in 2014–2015, membranes were placed in 45.5% (n = 91) of the patients. Osteotomy was a rare option for bone augmentation with three cases in 2010–2011 and two cases in 2014–2015. There was no statistical difference between the two time periods regarding different bone augmentation materials and technique.

### Statistical analysis

Statistical analysis was performed using SPSS for Windows release 21.0 (SPSS Inc., Chicago, IL, USA). The Chi-2 test was used to determine the differences between the two time periods (2010–2011, 2014–2015). Logistic regression analyses were performed for the prediction of adherence to national guidelines regarding antibiotic prophylaxis, or decision to either prolong the prophylaxis or refrain from its use. A *P*-value of < 0.05 was considered statistically significant.

## Results

### Antibiotic prescription patterns

The results show wide variation in antibiotic prescription pattern regarding type of compound, dose, and duration across the two time periods. However, there was no difference in the dentists’ postgraduate education level between the two time periods.

### Deviations from the national recommendation of antibiotic prophylaxis

The number of patients not treated with a single dose 2 g amoxicillin, as published in 2012 national recommendation, was high and comparable between the two time periods 75% (n = 150/200), 81.5% (n = 163/200), respectively, and the number of patients treated according to the national recommendation decreased between the two time periods. It was also found that there was an increase in the number of patients not receiving antibiotic prophylaxis from 11.5% (n = 23) in 2010–2011 to 27% (n = 54) in 2014–2015 (*P* = < 0.001) (Table 2).

**Table 2:** Antibiotic prescription pattern for patients undergoing bone augmentation procedure during the two studied time periods

Antibiotic prescription pattern	2010–2011 N (%)	2014–2015 N (%)	Chi-square	*P*-value
No Antibiotic prescribed	23 (11.5)	54 (27)	15.46	< 0.001
Antibiotic prescribed	177 (88.5)	146 (73)	15.46	< 0.001
According to the recommendation^a^	50 (25)	37 (18.5)	2.48	0.115
Not according to the recommendation^b^	127 (63.5)	109 (54.5)	6.2	0.012

^a^Treated according to the national recommendation: 2 g amoxicillin or, in case of penicillin allergy, 600 mg clindamycin 1 h preoperatively

^b^Not treated according to the national recommendation: any regimen other than the recommended compound and dose. Swedish guidelines published in October 2012, (Läkemedelsverket/Swedish Medical Products Agency, 2012)

### Deviations in compound and duration

**Fig. 1:**
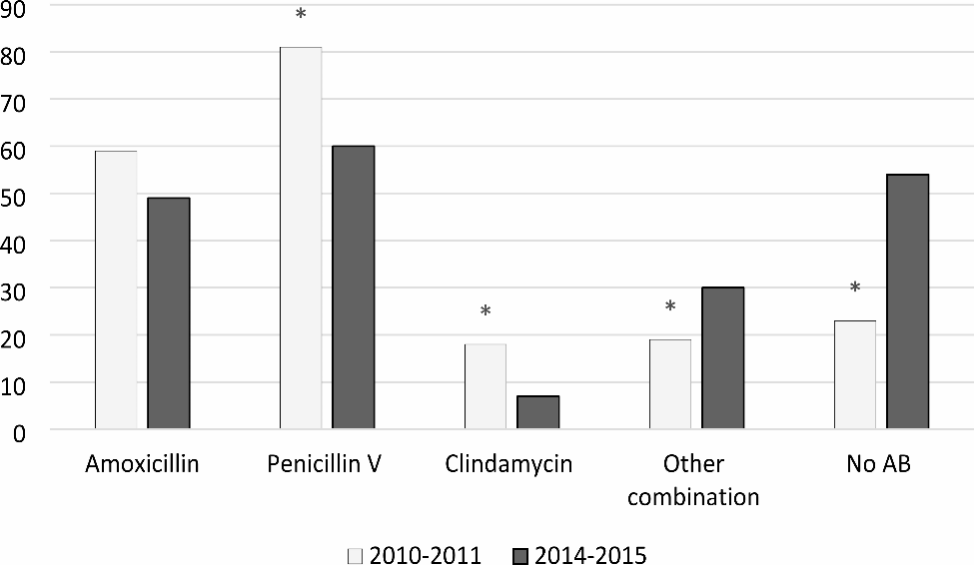
The number of patients receiving antibiotics in conjunction to bone augmentation procedures distributed between the two study periods and different compounds. AB, antibiotics. *, *P* < 0.05

The most common deviation from the recommendations for antibiotic prophylaxis was the use of phenoxymethylpenicillin which decreased from 40.5% (n = 81) in 2010–2011 to 30% (n = 60) in 2014–2015 (*P* = 0.026). Amoxicillin prescription was reduced slightly from 29.5% (n = 59) in 2010–2011 to 24.5% (n = 49) in the period of 2014–2015. A decrease (*P* = 0.0115) in usage of clindamycin was seen from 9% (n = 18) to 3.5% (n = 7) but an increase (*P* = 0.0435) in prescribing other antibiotic combinations from 9.5% (n = 19) to 15% (n = 30). The most common combination of compounds used was amoxicillin and phenoxymethylpenicillin (Fig. 1).

**Fig. 2:**
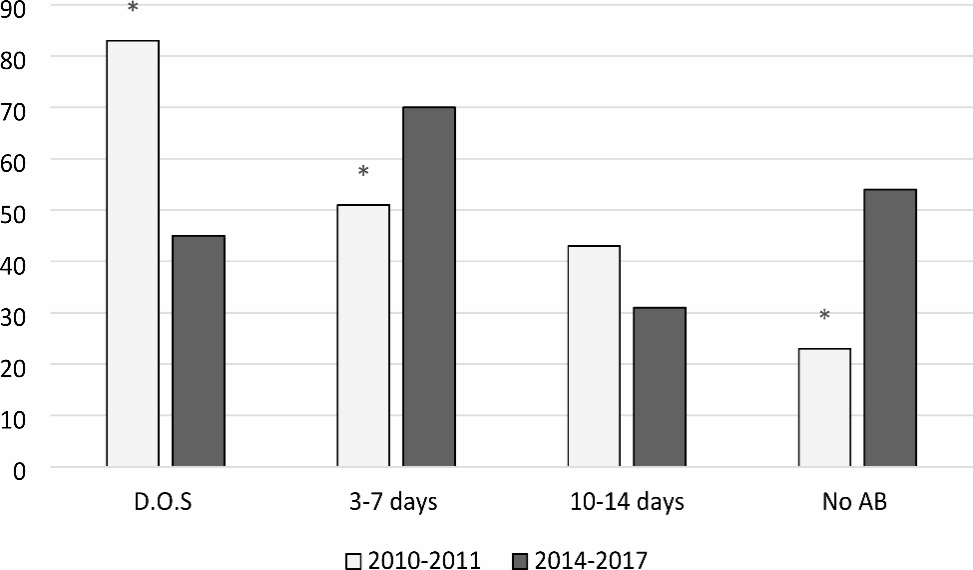
The number of patients distributed between different treatment durations in the two study periods. D.O.S., one or more doses of antibiotics on day of surgery; AB, antibiotics; *, *P* < 0.05

In 2010–2011, 47% (n = 83) of patients received antibiotics on the day of surgery, and there was a decrease (*P* = 0.0001) in numbers in 2014–2015 with only 31% (n = 45) of the patients receiving antibiotics on the day of surgery. Meanwhile there were increase (*P* = 0.038) in 3-7-days antibiotic prescriptions with 25.5% (n = 51) (2010–2011), and 35% (n = 70) in 2014–2015. Antibiotic prescription for a duration of 10–14 days decreased between the two time periods (Fig. 2). In summary, during the period of 2010–2011, 88.5% (n = 177) of the patients received antibiotics but only 25% (n = 50) of them were according to the recommendation and the rest 63.5% (n = 127) were extended antibiotic prescriptions, and 11.5% (n = 23) received no antibiotics. The numbers receiving antibiotics for period 2014–2015 were 73% (n = 146), also in this period only 18.5% (n = 37) followed the recommendation, 54.5% (n = 109) prolonged antibiotic treatment, while 27% (n = 54) received no antibiotics (Table 2).

According to the regression analyses there were no predictive factors for neither prolonged antibiotics prophylaxis prescription nor for not prescribing any antibiotics. However, there was a significant correlation between patients of male gender, occurrence of disease and prescribing antibiotics in alignment with the guidelines.

### Postoperative infection

The total rate of postoperative infection was 5.5% (22/400), 9 patients in 2010–2011 and 13 patients in 2014–2015 developed post-operative infections. All these complications were diagnosed within the first month after surgery. There was no difference between the two time periods for developing an infection (*P* = 0.382). From the nine patients who developed post-operative infections in 2010–2011, two were not given preoperative antibiotics, two received 2 g amoxicillin prophylaxis preoperatively, while five patients received other types of antibiotic prophylaxis. In 2014–2015, 11 patients were not prescribed antibiotics, one was treated with single dose 2 g amoxicillin prophylaxis according to the recommendation, and one patient received phenoxymethylpenicillin for seven days. The regression analysis shows a relationship between no antibiotic prescription, and the development of post-operative infection (*P* = 0.001). The twenty-two patients who developed post-operative infections were treated either locally by irrigation (n = 1), removal of the membrane (n = 1), or by removal of grafted bone or placed implant (n = 2). Systematic antibiotics were prescribed for most cases who developed an infection (8 cases in 2010–2011 and 13 cases in 2014–2015), with no difference regarding type and duration of prescribed antibiotic between the two time periods. Four patients were given a repeated antibiotic treatment.

## Discussion

This study shows that most of the patients who underwent bone augmentation procedures prior to dental implant treatment received prophylactic antibiotic with a wide variation in dose, duration, and type. Antibiotic prescription pattern comparing the two time periods was changed but differed from that recommended by the Swedish national authorities in 2012. An interesting observation in our study is that fewer patients received antibiotics during 2014–2015, but of those who received, a larger proportion was given a prolonged antibiotic prophylaxis. However, there was no correlation between prolonged prophylaxis and type of treatment, the patient’s health status, or the surgeon’s experience. Previous studies have concluded that dental practitioners who did not always follow clinical guidelines were not aware of the most current clinical guidelines regarding antibiotic prophylaxis despite the availability of these guidelines [13, 14]. Also lacking awareness of scientific evidence regarding appropriate and efficient antibiotic prescription might reduce the motivation to align with consensus recommendations. Moreover, phenoxymethylpenicillin was the most common antibiotic prescribed in the current study probably because it is recommended as the drug of choice for the treatment of dental infections in Scandinavia due to its narrow but relevant antimicrobial spectrum. Avoiding broad spectrum compounds gives the advantage of fewer side-effects and reduced ecologic shift in the normal microbiota and less risk for antibiotic resistance [15]. In our study, a dramatic inclination on not prescribing any antibiotic is observed. This might indicate that, implant surgeons attempt to be more restrictive and perhaps they became aware of the side effects of the antibiotics and developed more cautious prescription. A possible explanation is that the resident dentists’ training emphasized prudent antibiotics usage. This was evident in a previous study by Khalil et al., where the dentists were questioned about antibiotic knowledge during their education and found that most of the educational programs today emphasized on prudent antibiotic use [16]. Moreover, several studies conducted with dentists and medical doctors reported that educational initiatives played a key role in encouraging the rational and appropriate use of drugs [17, 18]. Interestingly, there seems to be a polarization in antibiotic prescriptions when comparing the two time periods, in 2014–2015 records showed that a restrictive group of surgeons gave no antibiotic prescription, while another group heavily overprescribed antibiotics. This might indicate that information and educational efforts to reduce antibiotic usage have been received differently between different clinicians or distributed in an uneven manner. The observed influence of patients’ gender on antibiotic prescription behavior was unexpected and calls for further studies to verify and explain.

5% of the patients developed postoperative infections following bone augmentation procedures. In the current study, the group of patients who did not receive antibiotics had significantly more infections compared to those given antibiotics. This could be explained by the fact that the kind of surgery performed (clean-contaminated surgery) had a 10–15% risk of infection where the risk is reduced to 1% with proper surgical technique and the use of prophylactic antibiotics [19, 20]. Since the current study wasn’t designed to primarily answer the question regarding postoperative infections, these results need to be interpreted with caution. However, it can be concluded that regardless of antibiotic regimen, infections rates are relatively low after these types of procedures. Dental implant installation with bone augmentation procedures was performed simultaneously in approximately half of the patients; otherwise, it was placed after a healing period following bone augmentation Von Arx and Buser have demonstrated that the best time for implant placement (simultaneous with the graft or after bone block placement) depends on the volume of the bone at the host site [21]. Simultaneous implant placement with the bone graft is suitable if the remaining bone allows for the correct positioning of the implant with primary stability [22]. However, others found that delayed implant placement will improve revascularization of the bone graft, which could lead to better bone-implant contact and secondary stability [23]. Therefore, the ideal time for implant and prosthesis installation needs to be individualized according to different bone grafts.

The present study constitutes the first step for a drug utilization review concerning antibiotic prescribing in patients who have undergone bone augmentation procedures in conjunction with dental implant treatment in Sweden. Stricter implementation efforts in this area are mandatory. Guidelines for antibiotic selection should be modified according to local factors, such as local resistant bacteria status and professional realities [24]. Post- and re-audits should be considered after the introduction of interventions designed to alter antibiotic prescribing practices [24]. The retrospective design is a limitation of the current study. However, there are limitations regarding the clinical validity of retrospective studies based on routine patient treatment (effectiveness study) as well as the more reliable and scientifically correct RCT study design [25]. Moreover, no patient records had to be excluded due to incomplete registration or notes rendering the data and statistics reliable. On the other hand, a retrospective design can prevent oblivious influence on prescription behavior because the surgeons are inherently blinded to the research question.

The results call for further nationwide studies on knowledge and attitudes towards the use of antibiotic prophylaxis in bone augmentation procedures. Randomized clinical trials are needed to provide guidelines based on solid scientific evidence.

## Conclusions

According to the present study the introduction of national recommendation of a preoperative single dose antibiotic prophylaxis before bone augmentation procedures led to an increase in the number of patients not receiving antibiotic prophylaxis and decrease of the group prescribed a single dose. Misuse or overuse of antibiotics seems to be common during bone augmentation procedures. Decision to adhere to guidelines regarding antibiotic use for these procedures does not seem to show gender equality. The results indicate a need for educational efforts and strategies for implementation of antibiotic prudence and awareness among surgeons.

## Data Availability

The data used to generate and support the findings of this study are available from the corresponding author upon request.
